# PROFILE OF DECEDENTS FROM A NATIONAL COHORT OF ASSISTED LIVING RESIDENTS

**DOI:** 10.1093/geroni/igac059.757

**Published:** 2022-12-20

**Authors:** Emma Belanger, Nicole Rosendaal, Xiao (Joyce) Wang, Joan Teno, Pedro Gozalo, David Dosa, Kali Thomas

**Affiliations:** Brown University, Providence, Rhode Island, United States; Brown University, Providence, Rhode Island, United States; Brown University, Providence, Rhode Island, United States; Oregon Health & Science University, Portland, Oregon, United States; Brown University, Providence, Rhode Island, United States; Brown University, Providence, Rhode Island, United States; Brown University, Providence, Rhode Island, United States

## Abstract

An increasing number of older adults reside in assisted living (AL) toward the end of life, and it remains unclear if this trend represents an additional place of care and end-of-life transition before eventual nursing home admission. Our objective was to examine the characteristics and healthcare utilization of AL residents who died during a two-year follow-up. We conducted a prospective cohort study of Medicare beneficiaries residing in large AL communities (25+ beds) in January 2017, and followed them until the end of 2018 using a variety of administrative healthcare claims data. The national population of Medicare beneficiaries in AL included 273,722 fee-for-service (FFS) beneficiaries, and 143,258 Medicare Advantage beneficiaries. From 2017 to the end of 2018, 23.7% of residents died. Of the 66,605 FFS Medicare beneficiaries who died during follow-up, 77.0% were 85 years old or older, 72.2% were diagnosed with Alzheimer’s disease and related dementia (ADRD) and 80.8% were diagnosed with heart failure or chronic obstructive pulmonary disease. Most FFS decedents (97.3%) resided in AL during their last 12 months of life, with 23.0% leaving AL before the last month of life. Half of FFS decedents died in AL, while another 24.1% died in a nursing home. AL communities represent an increasingly common place of end-of-life care for dying Medicare beneficiaries. These findings point to the need for state and federal policies to protect a growing population of frail and vulnerable AL residents.

